# *Socket2*: a program for locating, visualizing and analyzing coiled-coil interfaces in protein structures

**DOI:** 10.1093/bioinformatics/btab631

**Published:** 2021-09-08

**Authors:** Prasun Kumar, Derek N Woolfson

**Affiliations:** School of Chemistry, University of Bristol, Bristol BS8 1TS, UK; School of Chemistry, University of Bristol, Bristol BS8 1TS, UK; School of Biochemistry, University of Bristol, Bristol BS8 1TD, UK; Bristol BioDesign Institute, Life Sciences Building, University of Bristol, Bristol BS8 1TQ, UK

## Abstract

**Motivation:**

Protein–protein interactions are central to all biological processes. One frequently observed mode of such interactions is the α-helical coiled coil (CC). Thus, an ability to extract, visualize and analyze CC interfaces quickly and without expert guidance would facilitate a wide range of biological research. In 2001, we reported Socket, which locates and characterizes CCs in protein structures based on the knobs-into-holes (KIH) packing between helices in CCs. Since then, studies of natural and *de novo* designed CCs have boomed, and the number of CCs in the RCSB PDB has increased rapidly. Therefore, we have updated Socket and made it accessible to expert and nonexpert users alike.

**Results:**

The original Socket only classified CCs with up to six helices. Here, we report *Socket2*, which rectifies this oversight to identify CCs with any number of helices, and KIH interfaces with any of the 20 proteinogenic residues or incorporating nonnatural amino acids. In addition, we have developed a new and easy-to-use web server with additional features. These include the use of NGL Viewer for instantly visualizing CCs, and tabs for viewing the sequence repeats, helix-packing angles and core-packing geometries of CCs identified and calculated by *Socket2*.

**Availability and implementation:**

*Socket2* has been tested on all modern browsers. It can be accessed freely at http://coiledcoils.chm.bris.ac.uk/socket2/home.html. The source code is distributed using an MIT licence and available to download under the Downloads tab of the *Socket2* home page.

## 1 Introduction

α-Helical coiled-coil domains (CCs) are found widely in proteins from all kingdoms of life where they mediate protein–protein interactions and protein assemblies (Lupas and Bassler, 2017). CCs account for ≈5% of all known protein sequences (Rackham *et al.*, 2010). In structural terms, CCs comprise two or more α helices that wrap around each other in a rope-like fashion. The helices can be assembled in parallel or antiparallel arrangements, and as homo- or heteromeric complexes (Lupas *et al.*, 2017). In addition to their importance in biology, CCs are productive targets for *de novo* protein design (Korendovych and DeGrado, 2020; Woolfson, 2017, 2021), leading to applications in cell biology, synthetic biology and biotechnology (Beesley and Woolfson, 2019; Dawson *et al.*, 2019; Lapenta *et al.*, 2018).

The interactions between CC helices are tight and well-defined. These are known as knobs-into-holes (KIH) interactions as first proposed by Crick (1953). A ‘knob’ is defined as a side chain that projects from one helix and packs into a ‘hole’ formed by four side chains of an adjacent helix. These interactions are exploited by the program Socket (Walshaw and Woolfson, 2001) to identify CCs in the 3D structures of proteins deposited in RCSB PDB (Burley *et al.*, 2021). On this basis, Socket also identifies the underlying and usually 7-residue (heptad) repeats characteristic of CC sequences, assigning these to an a-to-g register (Lupas, 1996). Socket has been used by us to construct databases of CCs (Heal *et al.*, 2018; Moutevelis and Woolfson, 2009; Testa *et al.*, 2009) and tools for CC design and modelling (Wood and Woolfson, 2018; Wood *et al.*, 2017), and by others in a wide variety of CC-based research and applications (Walshaw and Woolfson, 2001). Socket has also been adopted and used widely, as evidenced by ≈300 and ≈400 citations in Web of Science and Google Scholar, respectively.

CC research has advanced considerably over the past 20 years, and there are now many more CC structures and sequences to explore and examine (Lupas *et al.*, 2017). Notably, an important class of CCs, the α-helical barrels (Woolfson *et al.*, 2015), has emerged that Socket does not identify. This issue is addressed by *iSocket*, a Python-based application programming interface (Heal *et al.*, 2018). Nonetheless, we felt that an updated Socket web server that is accessible to nonexpert users was needed. Therefore, we have upgraded Socket to *Socket2*, which can identify all CC architectures, and we have developed a *Socket2* webserver with a built-in visualizer and improved presentation of CC metadata that Socket generates.

## 2 Methods and implementation

*Socket2* recognizes KIH packing to identify CCs in proteins using structural criteria alone. For this, two files are required: (i) 3D coordinate file in PDB format (Burley *et al.*, 2021) and (ii) a *DSSP* output file (Joosten *et al.*, 2011; Kabsch and Sander, 1983). Details of the full methodology and parameters used are given in the original publication (Walshaw and Woolfson, 2001) and in the ‘Help’ tab of the *Socket2* home page.

### 2.1 Architecture

The *Socket2* webserver has three layers: the frontend, the backend and the software itself. The frontend is written in HTML, JavaScript and CSS. The home page provides various available options for running the program. Users can either provide a 4-character PDB ID or upload a .pdb/.cif/.mmcif file containing the 3D coordinates for a protein of interest. Any uploaded files are kept confidential and deleted within 12 h of upload. Users can also select the Socket parameters ‘packing cut-off’ and ‘helix extension’ from drop-down menus; otherwise, the default values of ‘7 Å’ and ‘0’, respectively, are used. The home page also provides background and related information under different tabs.

**Fig. 1.**
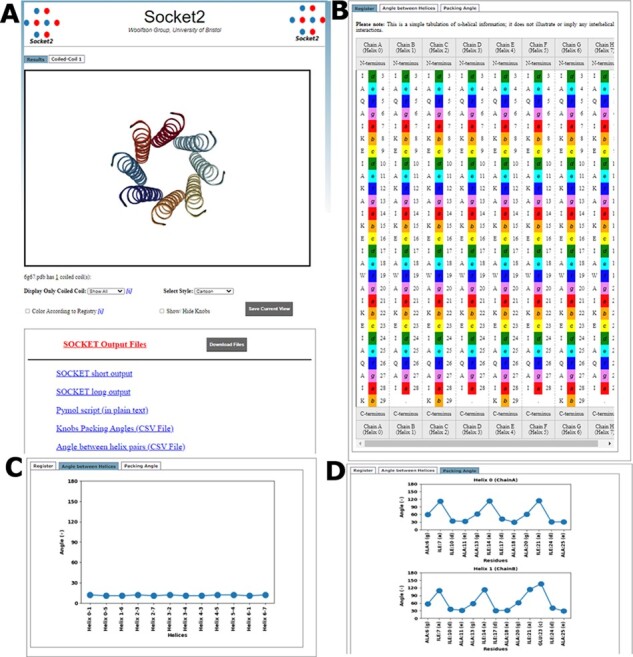
Overview of the pages of the *Socket2* webserver. (**A**) ‘Results’ page with NGL visualizer and links to different output files that can be downloaded as a zipped file. (**B**) Part of the tabulated information for sequences of participating α helices in identified CCs. Distributions of (**C**) angles between pair of helices of the CC and (**D**) packing angle of each identified knob residue. Example: the biological assembly from PDB ID 6G67 (Rhys *et al.*, 2018)

The frontend transfers the requests to the backend that runs DSSP and *Socket2*. The backend is written in CGI/Perl, HTML, JavaScript and CSS. Every successful run creates an output ‘Results’ page (Fig.  1A) with two parts: (i) a molecular visualizer and (ii) tabs detailing each identified CC. The webserver uses NGL Viewer (Rose *et al.*, 2018) to display the identified CCs. Sequences and heptad registers for each CC helix are also displayed (Fig.  1B). The webserver also uses Matplotlib (Hunter, 2007) to generate plots for helix–helix angles (Fig.  1C), and core-packing angles for the KIH interactions (Fig.  1D). Users can return to the home page to run further queries by clicking the *Socket2* icon.

### 2.2 Features

The *Socket2* web application has the following key features.

Biological assemblies: Some PDB entries have different asymmetric units and biological assemblies. The latter can be important for capturing full protein assemblies such as CCs. The webserver allows biological assembly to be used as the input by checking the box provided. This option is not available for uploaded files.

mmCIF files: In 2019, wwPDB made the use of mmCIF file format compulsory for the depositions of crystallographic methods. The webserver handles uploaded mmCIF files with *MAXIT* (https://sw-tools.rcsb.org/apps/MAXIT/index.html).

Modified residues: The MODRES record can be used to handle any modified residues or to rename a residue. The webserver searches for the presence of modified residues and, if not present, it adds a corresponding MODRES record to the input file allowing the *Socket2* program to run smoothly.

Visualization of CCs: Use of NGL Viewer allows an immediate inspection of any identified CCs, providing users an advantage over using the standalone version of *Socket2*. Each participating helix of the CC is initially displayed in different colours. Knob residues can be highlighted in ball-and-stick representation. Residues can then be rainbow-colour-coded according to their heptad register a-to-g.

Data representation: *Socket2* assigns a-to-g heptad registers to each chain of each identified CC. The webserver tabulates the name, number and heptad position for every residue (Fig.  1B), allowing quick inspection of sequence-to-structure relationships. Using Matplotlib, the webserver also plots interhelix angles for each CC (Fig.  1C), and core-packing angles for every knob residue (Fig.  1D).

Separate tabs for each CC: Structures may have one or more CCs. The webserver generates ‘Results’ tab for each CC to aid quick switching, inspection and analysis of these in large protein structures.

Metadata: The ‘Results’ tab also provides links to text files giving the detailed Socket outputs. a PyMol script allowing off-line visualization of the annotated CCs in PyMol (Schrödinger, 2021), and helix and core-packing angles (Fig.  1B). These will be particularly useful to those wishing to visualize and analyze sets of CC structures.

## 3 Applications

We anticipate that *Socket2* and data generated from it will be of use in gathering CC sequence statistics and structural parameters to improve sequence-to-structure relationships for CC-prediction (Ludwiczak *et al.*, 2019), modelling (Guzenko and Strelkov, 2018) and design (Korendovych and DeGrado, 2020; Woolfson, 2017, 2021). It will also facilitate the development and population of sequence and structural databases such as CC+ (Testa *et al.*, 2009), which, likewise, can be used to test CC-prediction algorithms and to develop rules for CC design. We envisage that the *Socket2* webserver will provide a useful gateway to such studies for experienced and new users alike.

## 4 Conclusions

Socket has been upgraded to *Socket2* to allow the identification of all possible CC architectures in multiple structure-file formats containing protein chains with proteinogenic or modified amino acids. The *Socket2* program is freely available to download under an MIT licence from http://coiledcoils.chm.bris.ac.uk/socket2/home.html. In addition, a user-friendly, interactive, and freely available webserver has been designed to run the program, and to allow quick visual inspection of the identified CCs and associated structural and sequence data. We anticipate that these tools with be useful to new and experienced cell, chemical, structural and synthetic biologist interested in natural and designed CC domains.
